# Timeliness of Enteric Disease Surveillance in 6 US States

**DOI:** 10.3201/eid1402.070666

**Published:** 2008-02

**Authors:** Craig W. Hedberg, Jesse F. Greenblatt, Bela T. Matyas, Jennifer Lemmings, Donald J. Sharp, Richard T. Skibicki, Arthur P. Liang, for the Enteric Disease Investigation Timeline Study Work Group

**Affiliations:** * University of Minnesota School of Public Health, Minneapolis, Minnesota, USA; † New Hampshire Department of Health and Human Services, Concord, New Hampshire, USA; ‡ Council of State and Territorial Epidemiologists, Atlanta, Georgia, USA; § Massachusetts Department of Public Health, Boston, Massachusetts, USA; ¶ Centers for Disease Control and Prevention, Atlanta, Georgia, USA

**Keywords:** public health, communicable disease control, epidemiology, surveillance, outbreaks, Escherichia coli O157:H7, Salmonella food poisoning, dispatch

## Abstract

We reviewed timeline information for a sample of *Salmonella* spp.*, Shigella* spp.*, Campylobacter* spp., and *Escherichia coli* O157:H7 cases and all confirmed foodborne outbreaks reported in 6 states during 2002. Increasing the timeliness of case follow-up, molecular subtyping, and linkage of results are critical to reducing delays in the investigation of foodborne outbreaks.

Timely reporting of foodborne diseases is necessary to identify persons at risk for exposure and to prevent additional cases in outbreak settings (*1*). The present study assesses time intervals for surveillance of foodborne diseases and investigation of outbreaks. Results establish baseline measures to evaluate foodborne disease surveillance systems and identify strategies for improvement (*2*–*4*).

## The Study

Data on case investigation timelines in 2002 were collected from records at state and local health departments and public health laboratories in each of 6 states for <100 *Salmonella* spp. isolates, <50 *Shigella* spp., *Escherichia coli* O157:H7, and *Campylobacter* spp. isolates, and for all foodborne outbreaks. Participating states included 1 with a large population (>6 million), 3 with a medium-sized population, and 2 with a small (<2 million) population from 5 different geographic regions. Two states received supplemental funding through FoodNet. Rules mandated reporting of diagnosed cases from physicians or clinical laboratories to local health departments (2 states), to the state health department (2 states), or to both (2 states). Cases were selected by systematically choosing every *n*th record on the basis of the number of cases reported and the number sampled.

For 1,319 cases, dates were collected for the following: onset of symptoms (873 [66%]), stool specimen collection (1,088 [82%]), culture result (633 [50%]), report to state or local health department (553 [42%]), submission of isolate to public health laboratory (882 [98%] of 899 isolates that were submitted), case interview (648 [49%]), and molecular subtyping by pulsed-field gel electrophoresis (PFGE) (634 of 635 isolates that were subtyped). Although stool culture result dates were recorded for 633 cases, most were for final culture results based on confirmation by the public health laboratory. Thus, initial culture result dates were available for 147 (11%) cases. For each case, intervals between milestones were calculated from the dates available.

For 112 outbreaks of foodborne disease, dates were collected for the following: implicated meal or event (100 [89%]), onset of symptoms of index case-patients (112 [100%]), first stool collection (65 [79%] of 82 outbreaks for which stool samples were collected), foodborne illness complaint or report of outbreak-related case to health department (99 [88%]), initiation of outbreak investigation activities (90 [80%]). For each outbreak, intervals were calculated from the dates available.

The median intervals from onset of symptoms to surveillance milestone events for individual cases were as follows (Table 1): collection of stool samples, 2–4 days; initial stool culture results, 5–8 days; case report to health department, 7–9 days; isolate submission to public health laboratory, 8–10 days. For case-patients who were interviewed, the median interval from onset of symptoms to interview was 12 days for *E. coli* O157:H7 cases, 14 days for *Salmonella* spp. and *Shigella* spp. cases, and 18 days for *Campylobacter* spp. cases. For isolates that were subtyped by PFGE, the median intervals from onset of symptoms to subtyping were 15 days for *E. coli* O157:H7, 18 days for *Salmonella* spp.*,* and 21 days for *Shigella* spp.

**Table 1:** Median number of days from onset of symptoms to specified timeline event for reported *Salmonella* spp., *Shigella* spp.*, Campylobacter* spp., and *Escherichia coli* O157:H7 infections, 6 US states, 2002

Timeline event	Median no. days after symptom onset			
	*Salmonella* spp.	*Shigella* spp.	*Campylobacter* spp.	*E. coli* O157
Collection of stool sample	4	2	3	3
Stool culture result	7	6	8	5
Case report from clinician to health department	9	8	9	7
Submission of isolate to public health laboratory	10	8	10	8
Case interview	14	14	18	12
PFGE* subtyping	18	21	Not routinely performed	15

*PFGE, pulsed-field gel electrophoresis.

A higher percentage of isolates were submitted to the public health laboratory in states where submission was required (98% for *Salmonella* spp. isolates, 100% for *E. coli* O157:H7) compared to states where submission was not required (75% for *Salmonella* spp. isolates, 80% for *E. coli* O157:H7). However, no difference was found between these states in length of time for isolates to be submitted.

Of 112 confirmed foodborne disease outbreaks, 83 (74%) had an etiologic agent confirmed by laboratory testing (Table 2) (*5*). Of 29 outbreaks that were not confirmed, norovirus was the suspected cause in 17 (59%) outbreaks, and toxigenic bacteria were suspected in 7 (24%) outbreaks. Median intervals from onset of symptoms to outbreak complaint or recognition were 1 day for bacterial toxins, 3 days for norovirus, 8 days for *E. coli* O157:H7 and *Campylobacter* spp.*,* and 16 days for *Salmonella* spp. (Table 2). Overall, 83 (74%) outbreaks were detected by a consumer complaint, 12 (11%) were detected by a healthcare provider, 11 (10%) were detected by PFGE cluster evaluation, and 6 (5%) were identified through an interview with an individual case-patient. Intervals from onset of symptoms to consumer complaint (median 3 days, range 0–21 days) or to report by healthcare provider (median 3 days, range 0–11 days) were similar. Outbreaks identified by case interview (median 11 days, range 6–16 days) or PFGE cluster evaluation (median 23 days, range 7–83 days) followed case surveillance timelines described above. The median interval from detection of the outbreak to the initiation of the first outbreak investigation step was 0 days (range 0–41 days) for all outbreaks.

**Table 2:** Median number of days from onset of symptoms to outbreak detection for outbreaks with confirmed etiology, 6 US states, 2002

Confirmed etiologic agent	No. (%) outbreaks with confirmed etiologic agent	Median no. days from onset of symptoms to outbreak detection (range)
*Salmonella* spp.	20 (24)	16 (2–83)
*Campylobacter* spp.	3 (4)	8 (7–9)
*Escherichia coli* O157:H7	4 (5)	8 (7–18)
Norovirus	44 (53)	3 (0–11)
*Bacillus cereus, Staphylococcus aureus,* and *Clostridium perfringens*	10 (12)	1 (0–3)

The median duration of exposure for all outbreaks with a confirmed etiologic agent was 1 day (range 1–21 days). However, 12 (29%) of 41 norovirus, 2 (67%) of 3 *E. coli* O157:H7, and 9 (75%) of 12 *Salmonella* spp. outbreaks occurred over multiple days. The median duration of multiday outbreaks was 4 days for norovirus (range 2–13 days), 5 days for *E. coli* O157/H7 outbreaks (range 5–6 days), and 10 days for *Salmonella* spp. outbreaks (range 3–21 days).

## Conclusions

The multiple steps between onset of a foodborne illness and its investigation by a public health agency result in delayed recognition of outbreaks caused by reportable enteric diseases. One important way to speed the detection of outbreaks is to encourage clinicians to immediately notify health departments when they suspect a patient is part of an outbreak. Since many outbreaks caused by *E. coli* O157:H7 and *Salmonella* spp. last multiple days, physician reporting concurrent with stool collection may provide opportunities for a public health intervention that could prevent outbreak-associated cases.

The speed with which clinical laboratories receive, process specimens, and report results varies by setting, agent, and location. The lack of detail available about these steps is an important limitation of this study. However, health departments generally receive reports from clinicians a median of 2 days after the culture result, and isolates are submitted to public health laboratories within 2–3 days of the initial culture result. These data suggest that improving physician and laboratory reporting practices and logistics could shorten the reporting timeline by 1 or 2 days for most cases.

Timeline elements directly under control of public health agencies include the interval from case report to interview and from submission of the isolate to subtyping by PFGE. Our results demonstrate more variability for these intervals than for earlier steps in enteric disease surveillance. In particular, *E. coli* O157:H7 infections appear to receive a higher priority than *Salmonella* spp.*, Shigella* spp.*, or Campylobacter* spp. infections. Half of *E. coli* O157:H7 cases but less than one fourth of *Salmonella* spp. cases were contacted by a local health department on the same day the report was received. In addition, outbreaks caused by *E. coli* O157:H7 were detected a median of 8 days sooner than outbreaks caused by *Salmonella* spp. Given the risk for hemolytic uremic syndrome after *E. coli* O157:H7 infections and the potential for person-to person transmission, such attention is warranted. Even so, the intervals from onset of symptoms to PFGE subtyping documented in the nationwide outbreak of *E. coli* O157:H7 infections associated with spinach demonstrated that little has changed across the public health system from 2002 to 2006 (*6*). This and other widespread outbreaks of *Salmonella* spp. infection reinforce the need to increase the timeliness of case follow-up, molecular subtyping, and the linkage of results between them that can reduce delays in the investigation of foodborne outbreaks (*7*).
